# The Combined Influence of Magnesium and Insulin on Central Metabolic Functions and Expression of Genes Involved in Magnesium Homeostasis of Cultured Bovine Adipocytes

**DOI:** 10.3390/ijms22115897

**Published:** 2021-05-31

**Authors:** Sandra K. Becker, Gerhard Sponder, Mansur A. Sandhu, Susanne Trappe, Martin Kolisek, Jörg R. Aschenbach

**Affiliations:** 1 Institute of Veterinary Physiology, Freie Universität Berlin, 14163 Berlin, Germany; s.becker@fu-berlin.de (S.K.B.); gerhard.sponder@fu-berlin.de (G.S.); mansoorsandhu@uaar.edu.pk (M.A.S.); susanne.trappe@fu-berlin.de (S.T.); 2 Department of Veterinary Biomedical Sciences, PMAS-Arid Agriculture University, Rawalpindi 46300, Pakistan; 3 Biomedical Center Martin, Jessenius Faculty of Medicine in Martin, Comenius University in Bratislava, Mala Hora 4D, 03601 Martin, Slovakia; Martin.Kolisek@uniba.sk

**Keywords:** magnesium, insulin, lipomobilization, adipocytes, cattle, adipose tissue, ketosis, fatty liver, magnesium-responsive genes, glycerol-3-phosphate dehydrogenase

## Abstract

At the onset of lactation, dairy cows suffer from insulin resistance, insulin deficiency or both, similar to human diabetes, resulting in lipolysis, ketosis and fatty liver. This work explored the combined effects of different levels of magnesium (0.1, 0.3, 1 and 3 mM) and insulin (25, 250 and 25,000 pM) on metabolic pathways and the expression of magnesium-responsive genes in a bovine adipocyte model. Magnesium starvation (0.1 mM) and low insulin (25 pM) independently decreased or tended to decrease the accumulation of non-polar lipids and uptake of the glucose analog 6-(N-(7-nitrobenz-2-oxa-1,3-diazol-4-yl)amino)-6-deoxyglucose (6-NBDG). Activity of glycerol 3-phosphate dehydrogenase (GPDH) was highest at 25 pM insulin and 3 mM magnesium. Expression of *SLC41A1* and *SLC41A3* was reduced at 0.1 mM magnesium either across insulin concentrations (*SLC41A1*) or at 250 pM insulin (*SLC41A3*). *MAGT1* expression was reduced at 3 mM magnesium. *NIPA1* expression was reduced at 3 mM and 0.1 mM magnesium at 25 and 250 pM insulin, respectively. Expression of *SLC41A2*, *CNNM2*, *TRPM6* and *TRPM7* was not affected. We conclude that magnesium promotes lipogenesis in adipocytes and inversely regulates the transcription of genes that increase vs. decrease cytosolic magnesium concentration. The induction of GAPDH activity by surplus magnesium at low insulin concentration can counteract excessive lipomobilization.

## 1. Introduction

During the transition period, i.e., three weeks prepartum to three weeks postpartum, dairy cows undergo a dramatic change in their physiological state due to the onset of lactation. At the onset of lactation, the energy demand increases abruptly and cannot be met by adequate intake of feed dry matter [1]. The consequence is a negative energy balance. To compensate the inadequate energy intake, the balance between lipogenesis and lipolysis in adipose tissue is shifted towards dominant lipolysis. This state of lipomobilization is linked to very low serum insulin concentrations, insulin resistance or both [2]. The decreased insulin sensitivity of adipose tissue and skeletal muscles [3] reduces glucose uptake by these tissues and consequently more energy metabolites, particularly glucose, are available for the mammary gland [4]. The reduction of insulin signals is physiological and necessary; however, any exaggeration predisposes the animals to metabolic diseases such as ketosis, fatty liver, milk fever, metritis, retained placenta or displaced abomasum [5,6]. Consequences are high veterinary costs [7] and losses in milk production and reproductive performance [8].

Previous experimental findings point to the fact that marginal Mg availability may promote ketosis in periparturient cows [9]. In contrast, dietary Mg provision far above requirement (0.61% of dry matter) increased fat-corrected milk production postpartum [10]. Studies in ewes with Mg supplementation above requirement suggest that such effects may originate from improved regulation of energy metabolism as demonstrated by decreased non-esterified fatty acids (NEFA) [11] and stabilized glucose concentrations in blood [12]. In contrast, experimentally induced hypomagnesaemia promoted lipolysis [13] and decreased both insulin responsiveness and insulin-mediated glucose disposal in sheep [14].

Magnesium interacts with numerous enzymes and substrates and thus plays a prominent role in energy metabolism, glucose homeostasis [15] and the regulation of triglyceride concentration in blood [16]. In this regard, a reciprocal influence of insulin and magnesium is particularly important. Magnesium is essential for phosphorylation of the insulin receptor and for the activity of downstream kinases. Adequate intracellular Mg concentrations are therefore essential for efficient insulin signaling [17]. At the same time, insulin promotes the retention of Mg in cells by reducing the activity of the main cellular magnesium extrusion system SLC41A1, a Na^+^/Mg^2+^ exchanger [18].

Insulin is thus a magnesiotropic hormone and, in turn, magnesium is an amplifier of the insulin signal. Consequently, adequate magnesium supply can increase the sensitivity towards insulin and has thus potential to alleviate exaggerated lipomobilization. The present research aimed to prove the hypothesis that supplementation of magnesium above physiological levels could improve insulin sensitivity of adipocytes and thereby reduce lipomobilization [19]. If valid, this could open new therapeutic options to prevent or alleviate metabolic diseases in dairy cows.

## 2. Results

### 2.1. Effect of Insulin and Mg on the Content of Non-Polar Lipids (Lipid/Nuclei Ratio)

To assess the combined influence of insulin and magnesium on cellular lipid content, the accumulation of intracellular non-polar lipids was measured by Nile-Red staining of adipocytes from six animals after 14 and 21 days in differentiation medium. To correct variations in cell density, the means of fluorescence signals for Nile red (lipid index) were divided by the means of DAPI fluorescence (nuclei index), yielding the lipid/nuclei ratio. Because of failed normality test, data were log-transformed before statistical evaluation.

**Figure 1:**
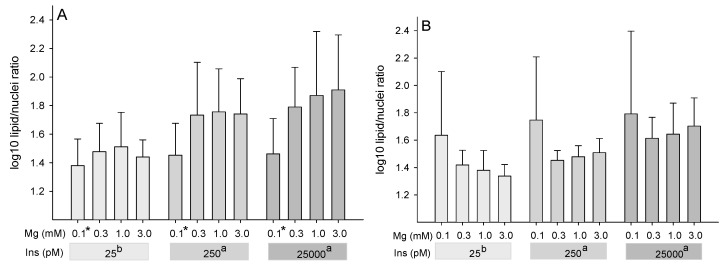
Quantification of non-polar lipids (ex. 475 nm/em. 570 nm) after 14 days (**A**) and after 21 days (**B**) in various differentiation media containing different concentrations of magnesium (Mg) or insulin (Ins). Non-polar lipids are presented relative to dsDNA signal (DAPI fluorescence of nuclei ex.358 nm/em. 461 nm). Results are given as means ± SD of six animals with two replicates per animal. * Asterisks indicate smaller LSM at 0.1 mM Mg compared to all other Mg concentrations (*P* ≤ 0.05). ^a,b^ Different superscript letters indicate different LSM for the factor insulin (*P* ≤ 0.05).

After 14 days of incubation in differentiation medium (Figure 1A), log-transformed lipid/nuclei ratio was affected by the factors insulin (*P* = 0.005) and Mg (*P* = 0.009) with a trend for Mg × insulin interaction (*P* = 0.078). The lowest insulin concentration (25 pM; LSM = 1.45 ± 0.051) showed the least accumulation of non-polar lipids per cell in comparison to a high physiological (250 pM; LSM = 1.67 ± 0.051) and a supraphysiological insulin concentration (25,000 pM; LSM = 1.76 ± 0.051), evidenced by a lower log-transformed lipid/nuclei ratio at 25 pM insulin (*P* ≤ 0.05 each). Across insulin concentrations, a significantly lower accumulation of non-polar lipids was observed at the lowest Mg concentration of 0.1 mM (LSM = 1.43 ± 0.056) in comparison to 0.3 mM (LSM = 1.67 ± 0.056), 1 mM (LSM = 1.71 ± 0.056) and 3 mM Mg (LSM = 1.70 ± 0.056) in the medium (*P* ≤ 0.05 each), where 1 mM was chosen to represent physiological Mg conditions [20].

**Figure 2:**
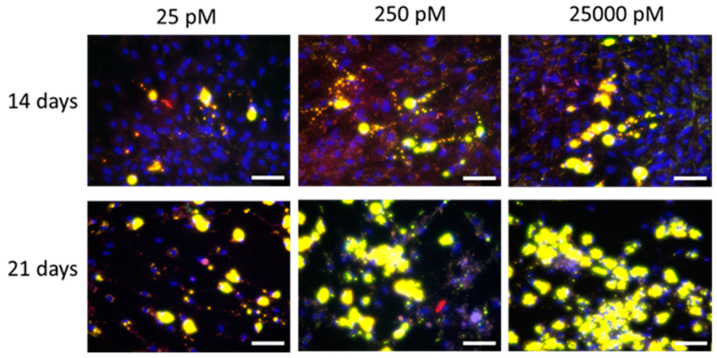
Representative Nile red staining of intracellular lipids in adipocytes after 14 days and 21 days of incubation using various insulin concentrations (25 pM, 250 pM and 25,000 pM) at a physiological magnesium concentration (1 mM). Nile red was imaged for total lipids at 515 nm/590 nm (ex./em.) and coded to red. Non-polar lipids were imaged at 475 nm/570 nm (ex./em.) and coded to green. A green-red overlay results in yellow color for lipid droplets. The blue color represents dsDNA of nuclei stained with DAPI and imaged at 358 nm/461 nm (ex./em.). Scale bar = 100 µm (20× objective).

After 21 days of culture (Figure 1B), the log-transformed lipid/nuclei ratio was affected by the factor insulin only (*P* = 0.001). A supraphysiological insulin concentration (25,000 pM; LSM = 1.69 ± 0.034) showed the highest log-transformed lipid/nuclei ratio in comparison to a high physiological (250 pM; LSM = 1.55 ± 0.034) and the low (25 pM; LSM = 1.44 ± 0.034) insulin concentration (*P* ≤ 0.05 each). An insulin concentration of 25 pM also tended to have lower log-transformed lipid/nuclei ratio compared to 250 pM (*P* = 0.055). No effects of Mg (*P* = 0.34) or Mg × insulin interaction (*P* = 0.20) were observed after 21 days in the differentiation medium. Representative images of lipid accumulation after 14 and 21 days at 1 mM Mg and different insulin concentrations are shown in Figure 2.

### 2.2. Influence of Insulin and Magnesium on Glucose Uptake of Adipocytes (Glucose/Nuclei Ratio)

The capacity for glucose uptake was examined using a 6-NBDG assay with cells from six animals after 14 and 21 days in differentiation medium. The fluorescence of 6-NBDG (glucose index) was normalized to DAPI fluorescence (nuclei index) as glucose/nuclei ratio in order to assess the capacity of glucose uptake per cell. Because of failed normality test, data were log-transformed before statistical evaluation.

**Figure 3:**
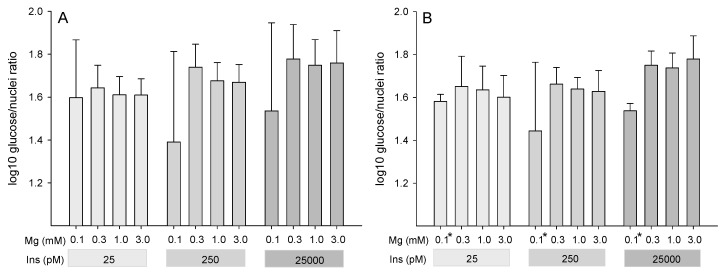
Assessment of the capacity for glucose uptake using the fluorescent glucose analog 6-NBDG (ex. 485 nm/em. 530 nm) after 14 days (**A**) and after 21 days (**B**) in various differentiation media containing different concentrations of magnesium (Mg) or insulin (Ins). DAPI fluorescence (ex. 358 nm/em. 461 nm) was used for normalization and results are expressed as log-transformed glucose/nuclei ratio. Results are means ± SD of six animals with two replicates per animal. * Asterisks indicate smaller LSM at 0.1 mM Mg compared to all other Mg concentrations (*P* ≤ 0.05).

After incubation for 14 days in differentiation medium, log-transformed glucose/nuclei ratio showed a rising trend with higher insulin concentrations (*P* = 0.057) but no effect of Mg concentration (*P* = 0.27) and no Mg × insulin interaction (*P* = 0.20; Figure 3A).

After 21 days of cultivation, the trend for effect of insulin concentration persisted (*P* = 0.10); however, log-transformed glucose/nuclei ratio was now significantly affected by the factor Mg (*P* = 0.007) with no Mg × insulin interaction (*P* = 0.25). The lowest tested Mg concentration (0.1 mM) had the lowest log-transformed glucose/nuclei ratio in comparison to all other tested Mg concentrations (*P* ≤ 0.05 each; Figure 3B).

### 2.3. Influence of Insulin and Magnesium on GPDH Activity of Adipocytes

Glycerol 3-phosphate dehydrogenase (GPDH) activity was investigated according to the manufacturer’s instructions with 1 × 10^6^ cells after 7 days of cultivation in various differentiation media. Least square means of five animals were statistically analyzed. The incubation at various insulin and Mg concentrations showed a statistically significant effect of insulin (*P* = 0.045) and Mg (*P* = 0.037) on the GPDH activity with no Mg × insulin interaction (*P* = 0.38; Table 1). The highest GPDH activity was measured at the lowest insulin concentration (25 pM) with a trend to decrease towards a higher physiological insulin concentration of 250 pM (*P* = 0.054). Cells incubated at the highest Mg concentration (3 mM) tended to have higher activity of GPDH compared to all lower Mg concentrations (0.1 mM, *P* = 0.052; 0.3 mM, *P* = 0.062 and 1 mM, *P* = 0.056), as seen in Table 1.

### 2.4. Influence of Insulin and Magnesium on the Expression of Magnesium-Responsive Genes

**Figure 4:**
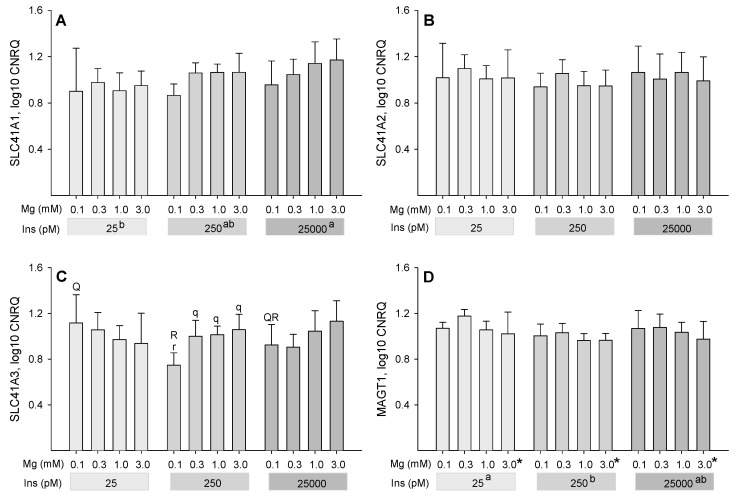

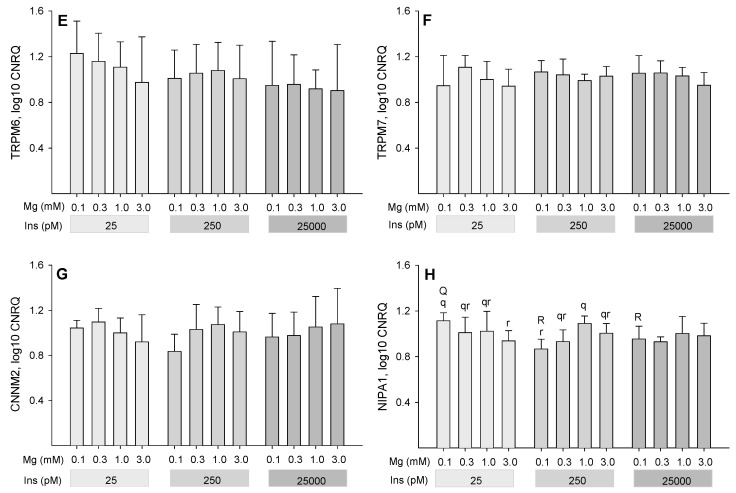
Relative mRNA expression (log10 CNRQ) of magnesium-responsive genes *SLC41A1* (**A**), *SLC41A2* (**B**), *SLC41A3* (**C**), *MAGT1* (**D**), *TRPM6* (**E**), *TRPM7* (**F**), *CNNM2* (**G**) and *NIPA1* (**H**) in bovine adipocytes. Cells were cultivated in differentiation media containing various concentrations of insulin (Ins) and magnesium (Mg) for 7 days. Results are given as means from six animals ± SD with three replicates. * Asterisks indicate smaller LSM at 3 mM compared to 0.3 mM Mg concentration (*P* ≤ 0.05). Superscript letters a and b indicate different LSM for the factor insulin (*P* ≤ 0.05). Lower case letters r and q indicate differences within a given insulin concentration (*P* ≤ 0.05) and Capital letters R and Q indicate differences within a given Mg concentration (*P* ≤ 0.05) whenever interaction between Mg × insulin was significant.

The log10-fold changes of the calibrated normalized relative quantity (CNRQ) of Mg-responsive genes of six animals were analyzed after 7 days of incubation in differentiation medium. No changes were found in the expression of the solute carrier family 41 member 2 (*SLC41A2*; Figure 4B), cyclin and CBS domain divalent metal cation transport mediator 2 (*CNNM2*; Figure 4G) and transient receptor potential cation channel subfamily M members 6 (*TRPM6*; Figure 4E) and 7 (*TRPM7*; Figure 4F).

The expression of the solute carrier family 41 member 1 (*SLC41A1*) was affected by both insulin (*P* = 0.021) and Mg concentration (*P* = 0.050) with no Mg × insulin interaction (*P* = 0.72; Figure 4A). Across Mg concentrations, *SLC41A1* expression was lower at the extremely low insulin concentration of 25 pM compared to the highest insulin concentration of 25,000 pM (*P* ≤ 0.05). Within the significant factor Mg, a trend for down-regulation was observed at the lowest Mg concentration (0.1 mM) compared to the highest Mg concentration (3 mM; *P* = 0.062).

No main effects of insulin or Mg were observed for solute carrier family 41 member 3 (*SLC41A3*). However, significant interaction was evident between Mg × insulin (*P* = 0.006; Figure 4C). The interaction identified that log10 CNRQ was lower when 0.1 mM Mg was compared to the other tested Mg concentrations at 250 pM insulin (*P* ≤ 0.05 each) and when 250 pM vs. 25 pM insulin concentrations were compared at 0.1 mM Mg concentration (*P* ≤ 0.05).

The Mg transporter 1 (*MAGT1*) was significantly affected by the factor insulin (*P* = 0.022) with an up-regulation at 25 pM insulin compared to 250 pM (*P* ≤ 0.05; Figure 4D). The effect of Mg concentration was also significant (*P* = 0.031), with a decrease in the LSM of log10 CNRQ when increasing Mg concentration from 0.3 mM to 3 mM (*P* ≤ 0.05). Interaction of Mg × insulin was not significant (*P* = 0.91) for *MAGT1* expression.

The main effects of Mg and insulin were not significant for the NIPA Mg transporter 1. However, a significant interaction between Mg × insulin was found (*P* = 0.011; Figure 4H). At the lowest insulin concentration of 25 pM, log10 CNRQ of *NIPA1* was highest at 0.1 mM and lowest at 3 mM Mg concentration (*P* ≤ 0.05). At an insulin concentration of 250 pM, however, log10 CNRQ of *NIPA1* was lowest at 0.1 mM and highest at 1 mM Mg concentration (*P* ≤ 0.05). As a consequence, log10 CNRQ of *NIPA1* was higher at 25 pM vs. 250 pM insulin (*P* ≤ 0.05) when compared within the 0.1 mM Mg treatments.

## 3. Discussion

Adipose tissue is an important energy reservoir. It plays a crucial role during the transition period when cows run into negative energy balance due to the onset of lactation [21]. Several adipose tissue deposits release their stored energy during this period with prominent contributions from visceral and subcutaneous adipose tissue. These two sources show gradual differences in their expression of anti- and prolipolytic receptors [22] and their proneness to inflammation [23]. However, their responses to changing energy status appear almost identical [22,23,24].

Insulin is a key signal that reports the energy status to adipocytes. It influences their metabolism, endocrine function and thereby whole-body energy homeostasis [6]. An additional role of Mg in adipocyte function is less well documented, especially in dairy cows. Appreciation of a combined role of Mg and insulin and, in particular, their functional interaction could provide novel prophylactic and therapeutic strategies against metabolic diseases of high-milk-producing cows in the period after calving. Therefore, the present study aimed at obtaining a better understanding of the complex interplay between lipid and carbohydrate metabolism and the role of insulin and Mg in cultured bovine adipocytes. We demonstrated earlier that cells cultured with our protocol express GLUT4, fatty acid binding protein FABP4, fatty acid synthase and peroxisome proliferator activated receptor PPARγ as key markers verifying their adipocyte functionality [25]. Using this model, we tested the effects of different concentrations of insulin and Mg in a two-factorial design on the accumulation of non-polar lipids, glucose uptake capacity, GPDH-activity and on the expression of various Mg-responsive genes in bovine adipocytes.

It is well documented that insulin has effects on adipocytes that are contrary to those of catecholamines. Insulin stimulates glucose uptake, transport of fatty acids into adipocytes, and lipogenesis [26]. Coherent with that general concept, insulin had a positive effect on the accumulation of intracellular non-polar lipids after 14 and 21 days of culture. Stimulation of lipid accumulation was maximized when proceeding from an extremely low insulin concentration of 25 pM to a high physiological insulin concentration of 250 pM and was not stimulated any further by increasing insulin concentration to supraphysiological levels (25,000 pM). This underlines both the functional relevance of our results and the appropriateness of the experimental model that represents adipocytes derived from ruminating cattle. The applied concentration range between 25 and 250 pM insulin largely mirrors the situation in vivo because, unlike humans and most other mammals, blood insulin concentration can be extremely low in postparturient dairy cows in severely negative energy balance. Weber et al. [27] reported that mean insulin concentrations dropped from ~250 pM at 3 weeks prepartum to ~25 pM at 2 weeks postpartum in dairy cows that developed fatty liver syndrome. Thus our in vitro model confirmed that high physiological insulin concentrations have adipogenic and protective functions against metabolic impairment, whereas a low insulin concentration caused lower incorporation of non-polar lipids and may eventually promote apoptosis of adipocytes [28].

The key intention of our study was to demonstrate an additional involvement of Mg in this process because it is known that insulin signaling is dependent on Mg [15]. In support of our hypothesis, extreme Mg starvation with only 0.1 mM Mg in the medium strongly impeded lipid accumulation at all insulin concentrations, at least, after 14 d of culture.

The adipocytes of our study had different substrate sources for lipid synthesis, glucose, acetic acid and serum lipids. It is established textbook knowledge that insulin stimulates the uptake of glucose through the glucose transporter GLUT4 and thereby stimulates fatty acid synthesis [29]. To investigate the influence of Mg on the insulin-dependent glucose uptake, a 6-NBDG assay was performed. The normalized 6-NBDG uptake in bovine adipocytes partly mirrored that of the lipid/nuclei ratio with a trend to increasing glucose uptake capacity with increasing insulin concentrations at day 14 and a depression of normalized 6-NBDG uptake at extreme Mg starvation (0.1 mM Mg) at day 21. The fact that insulin effects on glucose uptake capacity appeared subtler than insulin effects on lipid accumulation are coherent with the specific characteristics of adipose tissue of dairy cows. It has been shown that GLUT4 protein decreases in bovine adipose tissue already during postnatal development [30] and again with the start lactation [31]. Therefore, the limited insulin-responsiveness of glucose uptake in our study further emphasizes the applicability of our model because it resembles the situation of adult ruminants in vivo where GLUT1 appears to be a dominant glucose uptake pathway [32,33]. Irrespective of the subtle insulin effect, however, a positive effect of Mg on glucose uptake was evident. This effect might be mediated by Mg stimulation of the mammalian TOR complex 2 (mTORC2) [34], leading to GLUT1 phosphorylation and subsequently increased glucose uptake via this transporter [35]. This may imply that the contribution of adequate Mg supply to glucose uptake is not only dependent on the modulation of insulin action as has been proposed in human patients with diabetes mellitus [36,37], but may also include insulin-independent targets, especially in ruminants. Unfortunately, current knowledge about the Mg-insulin-glucose interplay is scarce in dairy cows. The few available studies mostly focused on the role of insulin for Mg status but not vice versa, the role of Mg for insulin effects [38,39].

Our third functional assay investigated insulin and Mg effects on the activity of GPDH, i.e., the enzyme that catalyzes reversible conversion of dihydroxyacetone phosphate and NADH into glycerol-3-phosphate and NAD^+^. The enzyme provides glycerol from carbohydrate metabolism to triglyceride synthesis [40]. Previously, it was shown that GPDH activity of bovine adipocytes is dependent on the presence of BSL, PPARγ agonist, dexamethasone and insulin [41]. In that previous study, complete omission of insulin from the culture medium reduced GPDH activity by 68% compared to the presence of 280 nM insulin [41]. That setup with complete omission of insulin was noticeably different from our setup where three levels of insulin treatment were compared. In our study, GPDH activity was influenced by both insulin and Mg with highest enzyme activities being observed at the lowest insulin concentration and the highest Mg concentration. Although the mechanisms behind that are not fully clear at present, this finding has important functional implications. Postparturient dairy cows regularly suffer from increased blood concentrations of non-esterified fatty acids that may cause fatty liver, ketosis and associated diseases [6]. Our present results suggest that Mg availability above physiological requirement may promote the synthesis of glycerol in adipocytes in the face of low plasma glucose and low plasma insulin concentrations and thus help sequestrating excessive serum lipids. Such scenario would help to ameliorate lipid accumulation in other tissues, e.g., fatty liver.

A final intention of the present study was to investigate the response of a set of so-called Mg-responsive genes to varying insulin and Mg concentrations. The Mg-responsiveness of many of these genes is known from previous experiments applying either dietary Mg restriction in mice or applying different Mg concentrations in cell culture experiments. For a comprehensive review see Kolisek et al. [42].

The expression of *SLC41A2*, *TRPM6*, *TRPM7* and *CNNM2* was not significantly affected by the availability of Mg and insulin in the present study. For the epithelial Mg channel TRPM6 [43] and the Mg-homeostatic factor CNNM2 [44], this fact may simply relate to the very low and variable expression observed in bovine adipocytes. For *TRPM7* this was not unexpected since the expression of *TRPM7* as a main entry mechanism for Mg^2+^ into cells seems to be constitutive [45]. Specifically for adipocytes (3T3-L1 cells), however, it was shown that TRPM7 channels contribute to adipogenesis and their deactivation impairs differentiation [46].

SLC41A1 as a Na^+^-dependent Mg exchanger is ubiquitously expressed [18] and its transport activity is stimulated by phosphorylation via cAMP-dependent protein kinase A (PKA) [47]. The latter results in higher Mg efflux and therefore a decrease in intracellular Mg concentration. Insulin signaling inherently counteracts PKA-dependent SLC41A1 activation and thus supports intracellular Mg retention [48]. In the present study, the mRNA expression of *SLC41A1* increased with increasing insulin concentrations and tended to increase with increasing Mg concentrations. A lower expression of SLC41A1 at low Mg concentration makes perfect sense because this would help to sequester Mg inside the cells when extracellular Mg availability is limiting. On the other hand, the inhibitory action of insulin on SLC41A1 function will also increase intracellular Mg levels [48], thus explaining a compensatory up-regulation of *SLC41A1* gene expression by high insulin concentrations to avoid excessive intracellular Mg accumulation, especially at concurrently high external Mg availability.

The transporter SLC41A3 has recently been characterized as a Na^+^-dependent Mg^2+^ extruder of the inner mitochondrial membrane [49]. In our experiments, the expression pattern of *SLC41A3* at 250 pM insulin mirrored the Mg effect on *SLC41A1*; therefore, equally suggesting that a downregulation of this transporter may rescue mitochondrial Mg concentration at low Mg availability. However, based on significant statistical interaction, this was obvious only as long cytosolic Mg concentration was protected by a high physiological insulin concentration of 250 pM. At 25 pM insulin, Mg deficiency combined with an insufficient insulin concentration seemed to stimulate the expression of the gene. If translated into functional SLC41A3, this might result in stronger extrusion of Mg^2+^ from mitochondria to sustain Mg availability in the cytosol [49,50].

The mRNA expression of *MAGT1* was up-regulated at lower Mg (0.3 mM vs. 3 mM) and lower insulin concentrations (25 vs. 250 pM) in the present study. The regulation of *MAGT1* expression was thus almost inverse to that of *SLC41A1*. This would be compatible with inverse regulation of and *SLC41A1* as a Mg efflux system and *MAGT1* as a Mg influx system, provided the latter postulate is correct [51,52].

An accepted electrogenic Mg influx pathway is NIPA Mg Transporter 1 (NIPA1). Previous studies showed that the *NIPA1* expression is dependent on the extracellular Mg concentration, with increased expression at low Mg concentrations [53,54]. In the present study, we identified the presence of *NIPA1* in bovine adipocytes with a significant Mg × insulin interaction. We detected up-regulation of *NIPA1* upon an undersupply (0.1 mM) of Mg in comparison to an oversupply (3 mM) only at the very low insulin concentration of 25 pM, confirming the results of Goytain et al. [53]. However, this pattern was inverted or neutralized at higher insulin concentrations, possibly indicating attempts to increase intracellular Mg retention upon increasing insulin availability as outlined in the discussion on SLC41A1 in a previous paragraph.

In conclusion, the present study investigated the effects of Mg and insulin on the differentiation of cultured bovine preadipocytes to mature adipocytes and lipogenesis. We demonstrated that insulin and Mg jointly promote these processes. An influence of insulin on glucose uptake capacity was only seen as a trend, which underlines the functional dominance of insulin-independent glucose uptake pathways that are inherent to our culture model and relevant for adipose tissue of adult cows. Mg influenced glucose uptake capacity of mature adipocytes positively and a supraphysiological Mg concentration of 3 mM increased GPDH activity, specifically at concurrently low insulin concentrations. The latter findings have the important pathophysiological implication that oversupply of Mg may have a potential to rescue the production of glycerol for triglyceride synthesis even in the case of low blood glucose and insulin concentrations. This could promote re-esterification of excessively circulating non-esterified fatty acids in adipocytes and thus counteract lipid injury to other organs, i.e., fatty liver and ketosis. The analysis of Mg-responsive genes further supported the important role of Mg in adipocytes during negative energy balance by demonstrating that certain genes that may rescue cytosolic Mg concentration during Mg starvation (*NIPA1* and *SLC41A3*) are specifically up-regulated at low insulin concentrations.

## 4. Materials and Methods

Bovine serum lipids (BSL) (Ex-Cyte), fetal bovine serum (FBS), penicillin-streptomycin, acetic acid, cell culture medium DMEM and Dulbecco´s phosphate-buffered saline (DPBS) were purchased from Merck Millipore (Darmstadt, Germany). N-2-Hydroxyethylpiperazine-N′-2-ethanesulfonic acid (HEPES), ascorbic acid, amphotericin B, biotin, bovine insulin, Nile red, trypan blue and D-glucose were obtained from Sigma Aldrich (Taufkirchen, Germany). 4′,6-diamidino-2-phenylindole (DAPI) was purchased from Roche (Grenzach-Wyhlen, Germany). Magnesium chloride solution was obtained from Honeywell Fluka™ (Taufkirchen, Germany). The 6-NBDG (6-(N-(7-nitrobenz-2-oxa-1,3-diazol-4-yl)amino)-6-deoxyglucose) was purchased from Life Technologies (Darmstadt, Germany). The Glycerol-3-Phosphate Dehydrogenase (GPDH) Activity Colorimetric Assay Kit was acquired from BioVision Inc. (Milpitas, CA, USA).

The NucleoSpin^®^ RNA kit was purchased from Macherey-Nagel GmbH & Co. (Düren, Germany). The iScript™ cDNA Synthesis Kit was obtained from Bio-Rad Laboratories GmbH (Munich, Germany). All primers and probes were synthesized by Eurofins Genomics (Ebersberg, Germany). Cell culture flasks were from Techno Plastic Products (Trasadingen, Switzerland), 24-well cell culture plates (CytoOne) were from Starlab (Hamburg, Germany), 96-well plates were sourced from Carl Roth (Karlsruhe, Germany) and 384-well plates were from Biozym Scientific GmbH (Hessisch Oldendorf, Germany).

### 4.1. Adipose Tissue Collection

Collection of adipose tissue was in accordance with the German legislation on animal welfare. All experiments were carried out using tissues from animals slaughtered for human consumption; i.e., no animals were specifically raised and killed to perform these experiments. Therefore, no animal use and care approval was required. Bovine subcutaneous adipose tissue was aseptically collected from the neck region (at the level of the 2nd to 3rd cervical vertebra) of exsanguinated Holstein cattle (median age, 9.5 months). Isolating stem cells from ~5 to 12 month-old cattle has the great advantage that these young animals have a larger pool of mesenchymal stem cells than older animals. On the other hand, cattle of this age are fully ruminating and thus represent the situation of lactating cows rather well. Explant culture and passage of pre-adipocytes was performed as described by Jurek et al. [25].

### 4.2. Induction and Differentiation of Adipocytes

For the evaluation of non-polar lipids and the measurement of glucose uptake, 1.5 × 10^4^ cells/mL were transferred into 24 well culture plates. For GPDH assay and RT-qPCR experiments, cells were seeded into T-75 flasks at a confluency of 30% and grown until 100% confluency. Cell passaging was implemented as described by Jurek et al. [25].

After reaching confluence, differentiation of pre-adipocytes into adipocytes was induced for 2 days as published by Jurek et al. [25]. For the subsequent experiments, differentiated adipocytes were kept in 12 different types of adipocyte differentiation media. Differentiation media were based on DMEM (without glucose, without Mg, with 4 mM stable L-glutamine and 15 mM HEPES) supplemented with D-glucose (10 mM), penicillin-streptomycin (100 U/mL and 100 μg/mL, respectively), amphotericin B (2.5 μg/mL), biotin (10 μM), ascorbic acid (113 μM), acetic acid (20 mM) and bovine serum lipids (5 μL/mL). This basic medium was complemented with 25 pM, 250 pM or 25,000 pM bovine insulin together with 0.1 mM, 0.3 mM, 1 mM or 3 mM magnesium (Mg^2+^) in a two-factorial design. Depending on the experiment and described below, cells were kept for 7, 14 or 21 days in the differentiation medium. The incubation was performed in a humidified atmosphere at 37 °C with 95% air and 5% CO_2_. The medium was replaced after every 48 h with 1 mL/well or 10 mL/T-75 flask.

### 4.3. General Procedures

#### 4.3.1. Measurement of Non-Polar Lipids

The content of intracellular, non-polar lipids was measured after 14 and 21 days in differentiation medium in 24-well plates. The protocol for staining, imaging and evaluation has been described by Sandhu et al. [55]. The fluorescence signal of Nile red (lipid index) was divided by the fluorescence signal of DAPI (nuclei index) to obtain the lipid/nuclei ratio, which represents the concentration of non-polar lipids corrected for cell density.

#### 4.3.2. Measurement of 6-NBDG Uptake

Uptake of 6-NBDG as surrogate for the uptake capacity for glucose was assessed after 14 and 21 days of cultivation in the above described differentiation media. 24 h before measurement, adipocytes were washed two times for 5 min with warm DPBS (without magnesium, without calcium). Subsequently, 1 mL/well DMEM (without glucose, with 4 mM L-glutamine), supplemented with D-glucose (5.5 mM), penicillin-streptomycin (100 U/mL and 100 μg/mL, respectively), amphotericin B (2.5 μg/mL) and HEPES (15 mM) was added. After 24 h, cells were washed two times for 5 min with warm DPBS (without magnesium, without calcium) in order to incubate the cells with 0.4 mL/well DMEM (without glucose, with 4 mM L-glutamine) supplemented with penicillin-streptomycin (100 U/mL and 100 μg/mL, respectively), amphotericin B (2.5 μg/ mL), HEPES (15 mM) and various insulin concentrations (25 pM, 250 pM or 25,000 pM) for 1 h. After removing the medium, cells were washed again two times for 5 min with warm DPBS (without magnesium, without calcium). DPBS was discarded and the cells were incubated with 0.4 mL/well of 6-NBDG (150 μM solved in glucose-free DMEM) at 37 °C for 30 min. After incubation, 6-NBDG was removed and cells were washed three times with ice cold DPBS (without magnesium, without calcium) with 0.5 mL/well DPBS remaining after the last washing step. The uptake of 6-NBDG was measured at 485/530 nm (excitation/emission; ex./em.) in a Multimode Plate Reader (PerkinElmer, MA, USA) in 24-well plates at 37 °C. Afterwards, DPBS was removed and the cells were incubated for 5 min with 0.5 mL/well DAPI (0.2 μg/mL in DPBS). DAPI fluorescence was measured at 358/461 nm (ex./em.) in the same Multimode Plate Reader.

The fluorescence signal of 6-NBDG (glucose index) was divided by the fluorescence signal of DAPI (nuclei index) to obtain the glucose/nuclei ratio, which represents the glucose uptake capacity corrected for variation in cell density.

#### 4.3.3. Glycerol 3-Phosphate Dehydrogenase (GPDH) Assay

Cultured adipocytes (7 days) were washed two times with warm DPBS (without magnesium, without calcium) and trypsinized in the same way as for passaging [25]. For stopping the trypsin reaction, cells were resuspended in DMEM (with 10% FBS and penicillin-streptomycin (100 U/mL and 100 μg/mL, respectively) and centrifuged at 350× *g* for 5 min at room temperature. The cell pellet was resuspended in 5 mL DPBS (without magnesium, without calcium). Subsequently, cells were stained with trypan blue and counted manually in a Neubauer cytometer. The measurement was conducted according to the manufacturer´s instruction with 1 × 10^6^ cells as duplicate in 96-well plates with an NADH standard curve and positive controls. The absorbance was measured at 450 nm in a Multimode Plate Reader (Tristar LB942, Berthold Technologies, Bad Wildbad, Germany) at 37 °C for 1 h.

#### 4.3.4. RNA Isolation and Quantitative Real-Time Polymerase Chain Reaction (RT-qPCR) of Magnesium-Responsive Genes

For RNA isolation, differentiated adipocytes were collected after 7 d as described by Jurek et al. [25] and immediately processed. RNA-isolation was performed by means of NucleoSpin^®^ RNA kit (Macherey-Nagel GmbH & Co., Düren, Germany) according to the manufacturer´s instructions. The integrity, purity and quantity of RNA was assessed by using the Bioanalyzer RNA 6000 Nano assay (Agilent Technologies, Santa Clara, CA, USA). All samples used had an RNA integrity number (RIN) ≥ 8. Aliquots of 1000 ng RNA of each sample were reverse transcribed to cDNA by using the iScript™ cDNA Sythesis Kit (Bio-Rad Laboratories GmbH, Munich, Germany) according to the manufacturer´s instructions in a Mastercycler™ Nexus Gradient (Eppendorf GmbH, Hamburg, Germany) in a one-step protocol (one cycle: priming at 25 °C for 5 min, reverse transcription at 46 °C for 20 min and inactivation of the transcriptase at 95 °C for 1 min).

Primers and probes for RT-qPCR are listed in Table 2. Three reference genes were used for normalization (β-actin [*ACTB*], ribosomal protein S19 [*RPS19*] and tyrosine 3-monooxygenase/tryptophan 5-monooxygenase activation protein zeta [*YWHAZ*]). RT-qPCR was carried out in a Viia 7 Real-Time PCR System (Thermo Fisher Scientific, Waltham, MA, USA) with Biozym Probe qPCR Kit separate ROX (Biozym Scientific GmbH, Hessisch Oldendorf, Germany). The program consisted of initial denaturation at 95 °C for 2 min, and 40 cycles with denaturation at 95 °C for 5 s, annealing and extension at 60 °C for 20 s.

Reactions were performed in final volumes of 9.9 µL, containing 4.5 µL of cDNA, 4.5 µL Biozym mastermix, 0.3 µL of each sense and antisense primers and probes as indicated in Table 2. All reactions were performed in triplicate. An inter-run calibrator (IRC) was utilized to correct for variations between the different runs and a no-template-control (NTC) was included for monitoring contamination and primer-dimer formations.

Thresholds were automatically calculated by the Viia 7 software. For data analysis, the software qbasePLUS (Biogazelle NV, Zwijnaarde, Belgium) was used to perform inter-run calibration, determining dilution series-based gene specific amplification efficiencies and testing for expression stability of reference genes. After normalization of Cq values with the respective reference gene(s), results were exported as calibrated normalized relative quantity (CNRQ) values which were used for statistical analysis.

### 4.4. Statistical Analysis

All presented data were statistically analyzed and all graphs plotted by using SigmaPlot 11.0 (Systat Software Inc., San Jose, CA, USA). Experiments to determine lipid/nuclei ratio and glucose/nuclei ratio were conducted in duplicates with cells from six animals. Analysis of GPDH activity was conducted in duplicates with cells from five animals. All multiple measurements were arithmetically pooled per animal and, if applicable, adjusted by subtracting blank values and matched to standard curves. All experiments were analyzed by two-way repeated measures analysis of variance (ANOVA) for the factors “animal”, “insulin” (25 pM, 250pM and 25,000 pM) and “Mg” (0.1 mM, 0.3 mM, 1 mM and 3 mM). Furthermore, possible interactions between Mg × insulin were tested.

The RT-qPCR data were retrieved from cells of six animals in triplicates, where each CNRQ value originated from triplicate RT-qPCR analysis. The RT-qPCR data sets were analyzed by two-way analysis of variance (ANOVA) with the effects of “insulin” (25 pM, 250pM and 25,000 pM), “Mg” (0.1 mM, 0.3 mM, 1 mM and 3 mM) and their two-way interaction.

If overall analysis of data showed statistical significance (*P* ≤ 0.05), differences between groups were identified by the Holm-Sidak post-hoc test. If normality test failed, data were log-transformed before statistical evaluation.

Data are presented as means ± standard deviation (SD) for individual groups or least square means (LSM) ± pooled standard error of means (SEM) for main effect data. Statistical significance was considered at *P* ≤ 0.05; trends are mentioned if 0.05 < *P* ≤ 0.1. The number of studied animals is given as *n*.

## Figures and Tables

**Table 1:** Least square means (LSM) of the glycerol-3-phosphate dehydrogenase (GPDH) activity in bovine adipocytes from five animals at various insulin (Ins in pM) and magnesium (Mg in mM) concentrations after 7 days of incubation.

Ins/Mg	0.1 MM	0.3 MM	1 MM	3 MM	LSM	SEM	
**25 PM**	1.58	1.45	1.26	3.24	1.88	0.170	**Factor** **insulin,** ***P* = 0.045** **Mg × insulin,** ***P* = 0.38**
**250 PM**	1.01	1.12	1.35	1.52	1.25	0.129	
**25,000 PM**	1.55	1.49	1.48	1.67	1.55	0.129	
**LSM**	1.38	1.35	1.36	2.14			
**SEM**	0.151	0.151	0.151	0.214			
**Factor Mg, *P* = 0.037**							

SEM is the pooled standard error of mean. Despite significant effects of insulin and Mg, Holm-Sidak post-hoc test could only identify trends towards highest GPDH activity at the lowest insulin and highest Mg concentration (*P* ≤ 0.1).

**Table 2:** Primer and probe sequences, applied concentrations (nM), as well as expected amplicon sizes (bp) and database accession numbers of magnesium-responsive genes and reference genes.

Gene	Sense Primer (5′-3′)	nM	Ampl. Size (bp)	Database Accession No.
	Anti-Sense Primer (3′-5′)			
	Probe			
***SLC41A1***	**TGGTGTTCCTCTATACCATCAG**	**1000**	186	NM_001206036.1
	TCAAGTACGGGATGGAGAAG	3000		
	ATGTAGAGCAGGATCAGCACCTGGAGCAGA	500		
***SLC41A2***	CTGCTTTTAGTGATACCTGGAC	500	178	NM_001205910.1
	TTCCTTTCCTCCAGAAATGATG	1500		
	TTGCTGTGGATCGCTGACTGGATG	150		
***SLC41A3***	CTTCTGCACTATTTCCAGCAC	1500	100	XM_024983333.1
	TCATCTCCAGGTTGCCCTTC	3000		
	TTCACGGAGATGAAGGACCTGCTGACCTTGG	500		
***MAGT1***	GCTCAATTTGTAGCTGAAACAC	500	124	NM_001244318.2
	CACACATTATCTTTCGCTTTCC	1500		
	ATGTGAAGCTGCTACATCTGACATGGATATTG	150		
***CNNM2***	GCTCCAGAATACTACCTCTACC	500	83	NM_001191172.1
	GCTTCTACTTCACTTTCCCC	1500		
	CGAAACAAACCTGTAGACTACTTCGTTCTCAT	150		
***NIPA1***	TCCCCGAAATCTGAGAGTGTG	1000	115	XM_002685192.6
	AGAAGATGAGCAGCAGCAGC	1500		
	TGGAGGAGAAGCTGACCAATCCAGTGTTTGTG	150		
***TRPM6***	ACAAACCATTCCCTACACTCC	500	125	XM_015472505.2
	CGTTGTTGTTGTTGTACTTCC	1500		
	TTGACCATCGAGAAGTATATGACGGGGGAG	150		
***TRPM7***	ATACAAGAGGGGAGTTACTGG	500	112	NM_001206166.3
	GGGCCAAAAACCATATCACAG	1500		
	CTGACCCATCTGTGATAAAGGCAGAAGAA	150		
***RPS19***	GGAAAAGGACCAAGATGGGG	500	136	NM_001037467.2
	CGAACGAGGCAATTTATTAACC	1500		
	ACAGAGAGATCTGGACAGAATCGCTGGACA	150		
***YWHAZ***	AGAGAGAAAATAGAGACCGAGC	500	144	NM_174814.2
	AGCCAAGTAGCGGTAGTAG	1500		
	CCAACGCTTCACAAGCAGAGAGCAAA	150		
***ACTB***	GCCAACCGTGAGAAGATGAC	500	124	NM_173979.3
	AGTCCATCACGATGCCAGTG	1500		
	CCAGATCATGTTTGAGACC TTCAACACCCCTGC	150		

## Data Availability

Data is contained within the article. The datasets analyzed during the current study are available from the corresponding author upon reasonable request.

## Abbreviations

6-NBDG	6-(N-(7-nitrobenz-2-oxa-1,3-diazol-4-yl)amino)-6-deoxyglucose
ACTB	β-actin
Akt (PKB)	protein kinase B
ANOVA	analysis of variance
BSL	bovine serum lipids
cAMP	cyclic adenosine monophosphate
cDNA	complementary DNA
CNNM2	cyclin and CBS domain divalent metal cation transport mediator 2
CNRQ	calibrated normalized relative quantity
DAPI	4′,6-diamidino-2-phenylindole
DMEM	Dulbecco’s Modified Eagle’s Medium
DPBS	Dulbecco’s phosphate-buffered saline
em.	emission
ex.	excitation
FBS	fetal bovine serum
GLUT	glucose transporter
GPDH	glycerol 3-phosphate dehydrogenase
HEPES	4-(2-hydroxyethyl)-1-piperazineethanesulfonic acid
Ins	insulin
IRC	inter-run calibrator
IRS	insulin receptor substrate
LSM	least square means
MAGT1	magnesium transporter 1
NAD^+^	nicotinamide adenine dinucleotide
NADH	reduced nicotinamide adenine dinucleotide
NIPA1	Nipa magnesium transporter 1
NTC	no template control
PDE3b	phosphodiesterase 3b
PDK1	phosphoinositide-dependent kinase-1
PI3K	phosphoinositide 3-kinase
PKA	protein kinase A
RIN	RNA integrity number
RNA	ribonucleic acid
ROX	carboxy-X-rhodamine
RPS19	ribosomal protein S19
RT-qPCR	reverse-transcription quantitative polymerase chain reaction
SD	standard deviation
SEM	standard error of mean
SLC	solute carrier family
STT3B	STT3 oligosaccharyltransferase complex catalytic subunit B
TRPM	transient receptor potential cation channel subfamily M
YWHAZ	tyrosine 3-monooxygenase/tryptophan 5-monooxygenase activation protein zeta
